# Translational Intracerebral Hemorrhage Research: Has Current Neuroprotection Research ARRIVEd at a Standard for Experimental Design and Reporting?

**DOI:** 10.1007/s12975-020-00824-x

**Published:** 2020-06-05

**Authors:** Lane J. Liddle, Shivani Ralhan, Daniel L. Ward, Frederick Colbourne

**Affiliations:** 1 Department of Psychology, University of Alberta, Edmonton, Alberta Canada; 2 Neuroscience and Mental Health Institute, University of Alberta, Edmonton, Alberta T6G 2E9 Canada

**Keywords:** Neuroprotection, Intracerebral hemorrhage, Translational research, Reproducibility, Animal models, Systematic review, Experimental design, Experimental reporting

## Abstract

One major aim of preclinical intracerebral hemorrhage (ICH) research is to develop and test potential neuroprotectants. Published guidelines for experimental design and reporting stress the importance of clearly and completely reporting results and methodological details to ensure reproducibility and maximize information availability. The current review has two objectives: first, to characterize current ICH neuroprotection research and, second, to analyze aspects of translational design in preclinical ICH studies. Translational design is the adoption and reporting of experimental design characteristics that are thought to be clinically relevant and critical to reproducibility in animal studies (e.g., conducting and reporting experiments according to the STAIR and ARRIVE guidelines, respectively). Given that ICH has no current neuroprotective treatments and an ongoing reproducibility crisis in preclinical research, translational design should be considered by investigators. We conducted a systematic review of ICH research from 2015 to 2019 using the PubMed database. Our search returned 281 published manuscripts studying putative neuroprotectants in animal models. Contemporary ICH research predominantly uses young, healthy male rodents. The collagenase model is the most commonly used. Reporting of group sizes, blinding, and randomization are almost unanimous, but group size calculations, mortality and exclusion criteria, and animal model characteristics are infrequently reported. Overall, current ICH neuroprotection research somewhat aligns with experimental design and reporting guidelines. However, there are areas for improvement. Because failure to consider translational design is associated with inflation of effect sizes (and possibly hindered reproducibility), we suggest that researchers, editors, and publishers collaboratively consider enhanced adherence to published guidelines.

## Introduction

Intracerebral hemorrhage (ICH) is a deadly stroke subtype, accounting for ~ 15% of all strokes [1]. ICH affects ~ 5 million people each year worldwide. Of those, ~ 3 million will die within 1 year, and only 12–39% of survivors will regain functional independence [2]. These statistics are concerning given that the incidence of ICH rose globally by 47% between 1990 and 2010, yet the rates of death and disability were unchanged during this time [1, 3]. Rates of disability following ICH are quite high, and we lack effective neuroprotective interventions. Those are expected to arise from rigorous and coordinated evaluation at the preclinical and clinical levels (the so-called translational pipeline) [4].

Translational experimental design is the adoption of a research plan that may advance a novel therapy from bench to bedside [5]. Translational design evolves, changing as the therapeutic approach becomes better understood. For example, safety and exploratory studies are often the first steps, whereas more rigorous (and costly) testing elements are added later as we progress towards translation (e.g., varying age and/or sex; using preclinical randomized controlled trials) [5, 6]. In sum, translational research uses information from basic and clinical sciences to advance therapies to the clinical realm.

In stroke neuroprotection, reproducibility and bias reduction are key issues [7, 8]. To address these, preclinical stroke researchers are encouraged to follow the STAIR and RIGOR guidelines [6, 9–11]. These contain widely adopted experimental design elements that preclinical scientists can use to produce more meticulous research [11–13]. These elements are blinding, randomization, a priori sample size calculations, explicit a priori statement of inclusion and exclusion criteria, and replication in multiple laboratories, to name a few [9, 14]. Although seemingly trivial, these experimental design components may have striking implications for translation. For example, previous studies have shown that more strict adherence to translational design associates with decreased efficacy (i.e., smaller effect sizes) of putative neuroprotective agents under study [15]. Thus, failing to use randomization and blinding could lead to bias in experimental neuroprotection research.

There are no agreed-upon definitions for neuroprotection. However, it is often described as an intervention that can preserve brain structure and/or function [15–19]. In the clinic, neuroprotection is commonly measured behaviorally (e.g., using the modified Rankin scale) [19–21]. Conversely, preclinical ischemia studies tend to measure infarct volume to gauge neuroprotection [19]. In preclinical ICH studies, the most common endpoints are behavior and brain water content (edema) assessments [22]. Although edema is commonly used preclinically, the relationship between edema and clinical outcomes is unclear, though recent studies suggest that peri-hematoma edema expansion is an independent predictor of ICH outcomes [23–25]. Thus, simple preclinical edema assessments (one-time wet-dry weight measurements) may not equivalently predict behavioral outcome as well as clinical edema markers (serial neuroimaging of edema expansion). The STAIR guidelines suggest that long-term (> 2 weeks post-stroke) behavioral and histological assessments should be performed in preclinical stroke studies, with emphasis placed on functional outcomes. This is especially important in the context of ICH, as post-ICH secondary injury can occur over several weeks [26, 27].

Similar to designing experiments using the STAIR and RIGOR guidelines, researchers can follow the ARRIVE guidelines to report important aspects of an experiment during publication, and ARRIVE is available in a checklist format which can be easily published as supplemental material [28]. The ARRIVE guidelines were developed with the rationale that improved reporting of published literature could lead to improved reproducibility, by fully characterizing a study. Aside from completely reporting analyses and results, ARRIVE outlines critical information required to interpret and replicate the study, for example, drug doses, routes, and timing of administration; animal age, weight, strain, sex, sample sizes, and method of their determination; and bias reduction measures (e.g., randomization and blinding), among others. Not only are the ARRIVE guidelines important for replicability, but this information is critical for research interpretation and synthesis (e.g., during meta-analyses, study quality assessments).

Given that experimental design is seen as critical to research translation, the present review aims to provide a snapshot of the quality of experimental design in ICH neuroprotection research and some considerations for researchers. Using the STAIR and ARRIVE guidelines as a framework to analyze experimental design and reporting, we sought to systematically investigate the reporting of in vivo preclinical experiments from the past 5 years of ICH neuroprotection research. Specifically, given that published guidelines suggest long-term behavioral and histological assessment, one goal of this study was to analyze the type and timing of behavioral and histological assessments in current preclinical ICH research.

## Methods

**Fig. 1:**
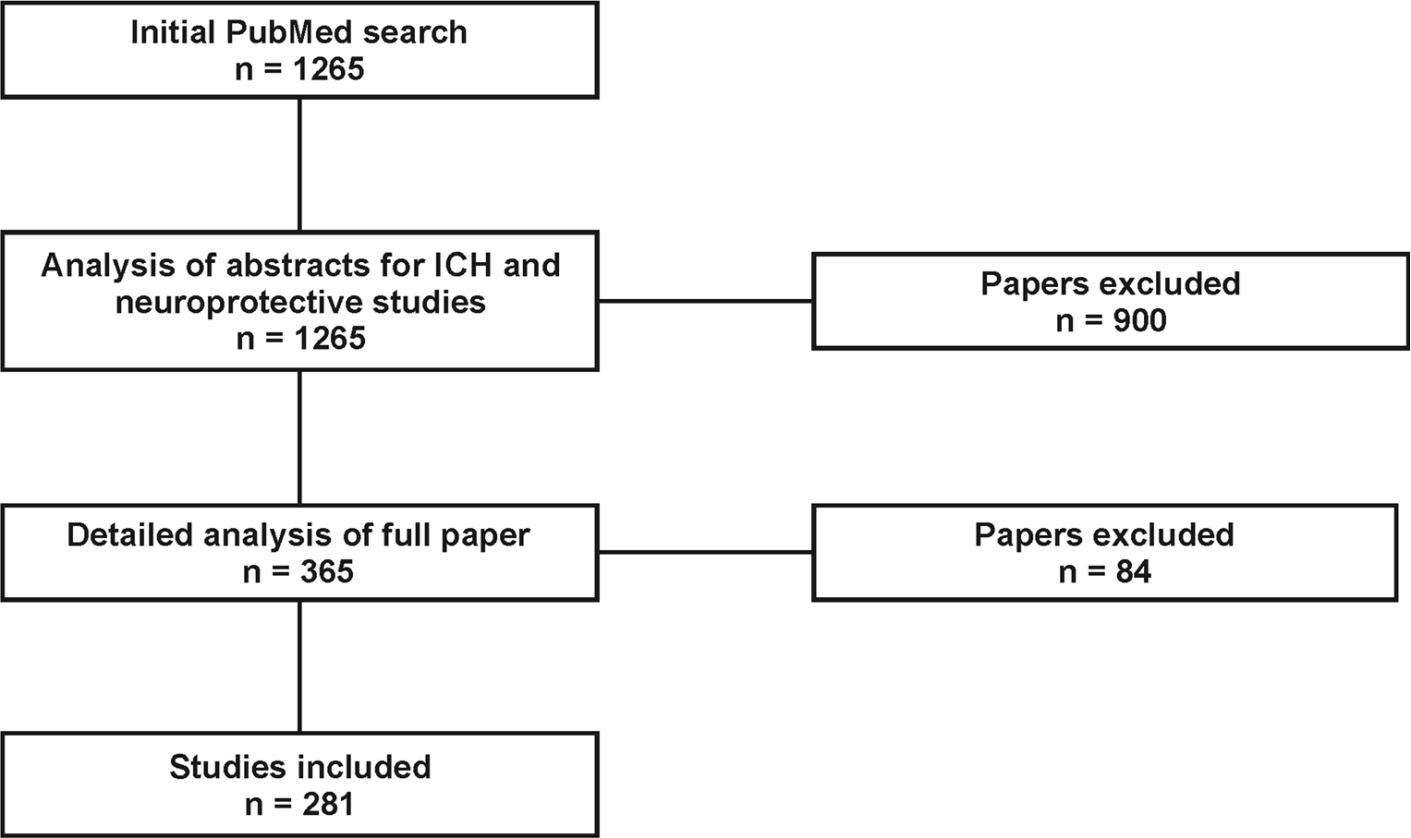
Overview of articles revealed from PubMed search and total studies selected for analyses. The inclusion criteria involved intracerebral hemorrhage (ICH) studies that evaluated a neuroprotective or cell-saving therapy. The search criteria included the following terms: “(animal OR rodent OR rat OR mouse)” AND “(intracerebral hemorrhage OR intraparenchymal hemorrhage OR intrastriatal hemorrhage)” NOT “(ischemia OR subarachnoid hemorrhage OR traumatic brain injury OR middle cerebral artery occlusion model)”

We searched the PubMed database from January 2015 to June 2019. The search criteria were made with the combination of the following terms: “(animal OR rodent OR rat OR mouse)” AND “(intracerebral hemorrhage OR intraparenchymal hemorrhage OR intrastriatal hemorrhage)” NOT “(ischemia OR subarachnoid hemorrhage OR traumatic brain injury OR middle cerebral artery occlusion model).” Using these search criteria, abstracts and titles of 1265 articles were returned and screened for eligibility. The inclusion criteria comprised only in vivo experimental ICH studies written in the English language. Of the 1265 articles returned in our search, 281 met inclusion criteria and were analyzed for experimental design characteristics (e.g., endpoints used, model descriptions, and alignment with STAIR and ARRIVE guidelines), and the venue of publication was also extracted, in addition to whether the venue required mandatory reporting in accordance with published guidelines. A flowchart of the search results can be found in Fig. 1. Our analysis focused exclusively on studies with a clearly defined ictus and intervention. Therefore, spontaneous ICH models were also excluded (though these models were used infrequently). Finally, as the main focus of this analysis was centered around adult ICH models, intrauterine and neonatal models were excluded from our analysis.

## Results

### Experimental Design Reporting

**Fig. 2:**
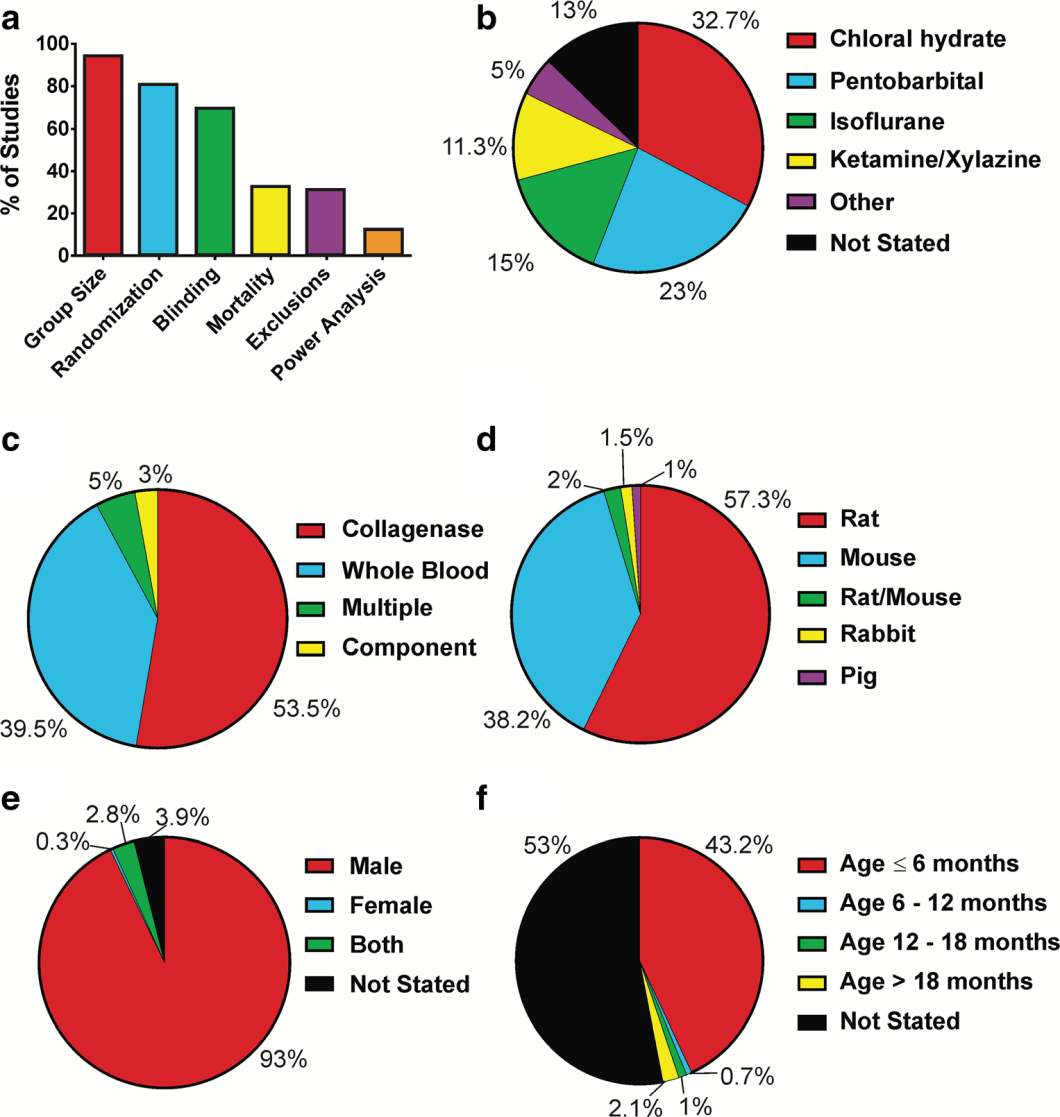
Analysis of experimental design. **a** Proportion of studies reporting key components of translational design. **b** Distribution of anesthetic use across all studies analyzed. **c** Analysis of ICH models used in current ICH research. **d** Species of animals used in current ICH neuroprotection research. **e** Sex of animals used in ICH neuroprotection research. **f** Proportion of studies using animals within particular age groupings

In our analysis of experimental design, we found that 94% of studies clearly report the number of animals used per group. We also found that 12.5% of studies detailed a priori how many animals per group were deemed necessary to detect an expected effect. A majority of authors now report use of randomization or blinding in their publications. An overview of experimental design reporting can be found in Fig. 2a.

### Characteristics of Animal Models Used in ICH Neuroprotection Research

In our evaluation of ICH neuroprotection research, we characterized most aspects of the animal ICH models used. We found that most studies make use of chloral hydrate, sodium pentobarbital, or isoflurane anesthesia (Fig. 2b). We also found that about 50% of the published studies used the collagenase model of ICH [29]. About 40% of studies used the blood infusion model of ICH [30, 31]. Remaining studies used blood component models (e.g., thrombin or iron) or multiple models (e.g., collagenase and blood infusion model or blood infusion model and thrombin model; Fig. 2c). In terms of the animal species used in preclinical research, about 60% of studies use rats, and 38% of studies use mice (Fig. 2d). Pigs and rabbits are uncommon ICH models, and few studies are performed using multiple animal species.

#### Weight and Age Reporting

Forty-seven percent of studies reported the age of animals used in the study. Eighty-three percent of studies stated the weight of the animals used. Only 34% of studies reported both the weight and age of the animals used in the study.

#### Sex Differences in ICH Neuroprotection Research

Of the 281 total studies, 270 reported the biological sex of the animals used. Of these studies, we found that about 96% used male rats and only about 3% of studies were conducted using female rats (Fig. 2e**)**.

#### Animal Health Status

Overall, we found 16 studies that used animals with advanced age or comorbid conditions. Three studies used hypertensive rats, 4 studies used rats with hyperglycemia, and 9 studies used aged (age > 1 year) rats. A breakdown of animal age categories used in current preclinical ICH research is depicted in Fig. 2f. In sum, of the 281 experimental neuroprotection studies, about 5% used animals with altered health status.

### Neuroprotective Intervention Characteristics

**Fig. 3:**
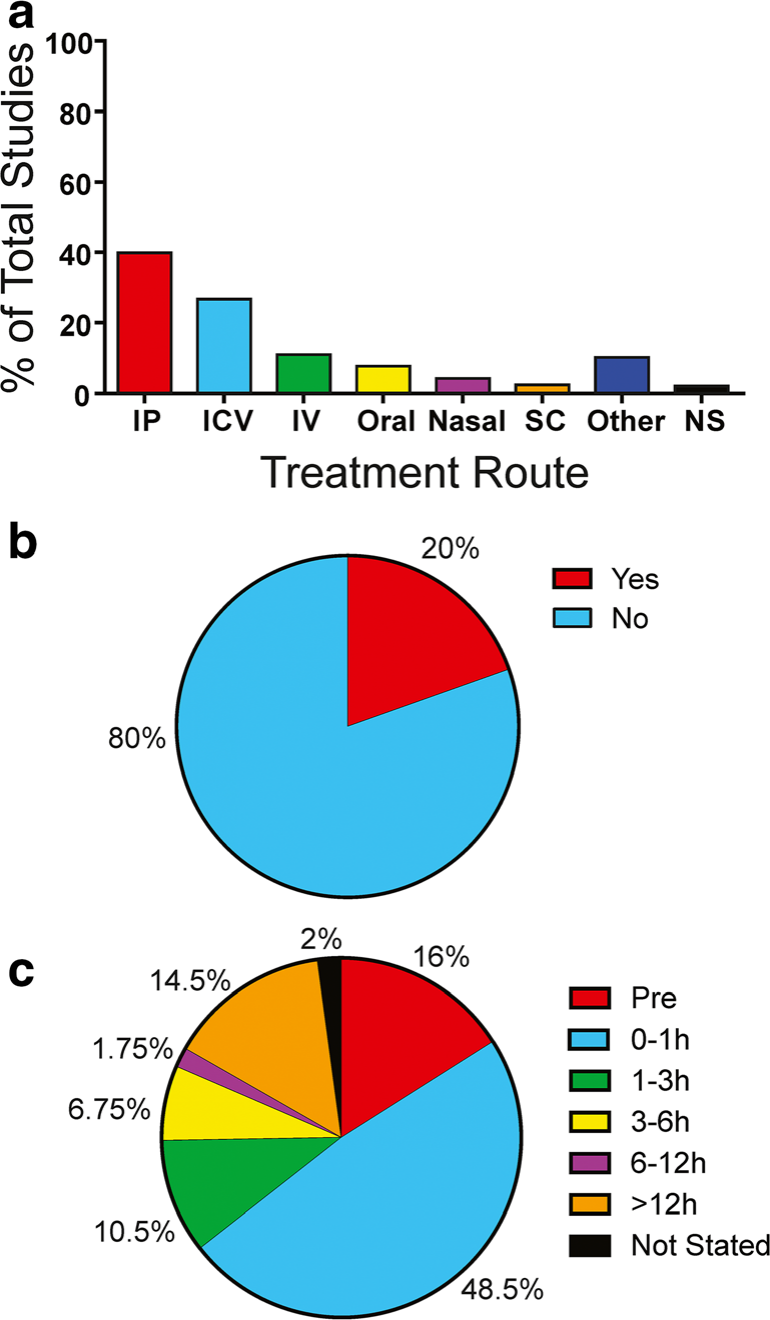
Assessment of ICH neuroprotection intervention parameters. **a** Breakdown of treatment administration route across studies. **b** Evaluation of whether treatment efficacy was shown in a dose-response fashion. **c** Longest treatment delay in ICH neuroprotection studies

To better characterize ICH neuroprotection research, we evaluated how neuroprotective interventions are delivered, whether a dose-response relationship was shown, whether intervention timing was considered, and the latest intervention delay within each study. We found that intraperitoneal injection was the most common route of administration, followed by intracerebroventricular administration (Fig. 3a). Few studies (~ 20%) did dose-response assessment (Fig. 3b). Only ~ 7% of studies varied the delay between ICH and treatment administration. Thus, most studies treated animals at only one time after ICH, and most treated within the first hour (Fig. 3c). Remarkably, 1 in 6 ICH neuroprotection studies used pre-ICH treatment. Only ~ 16% of neuroprotective interventions were given after a 6-h or longer delay. Finally, no study compared treatment efficacy across a range in hemorrhage volumes.

### Tissue Endpoints

**Fig. 4:**
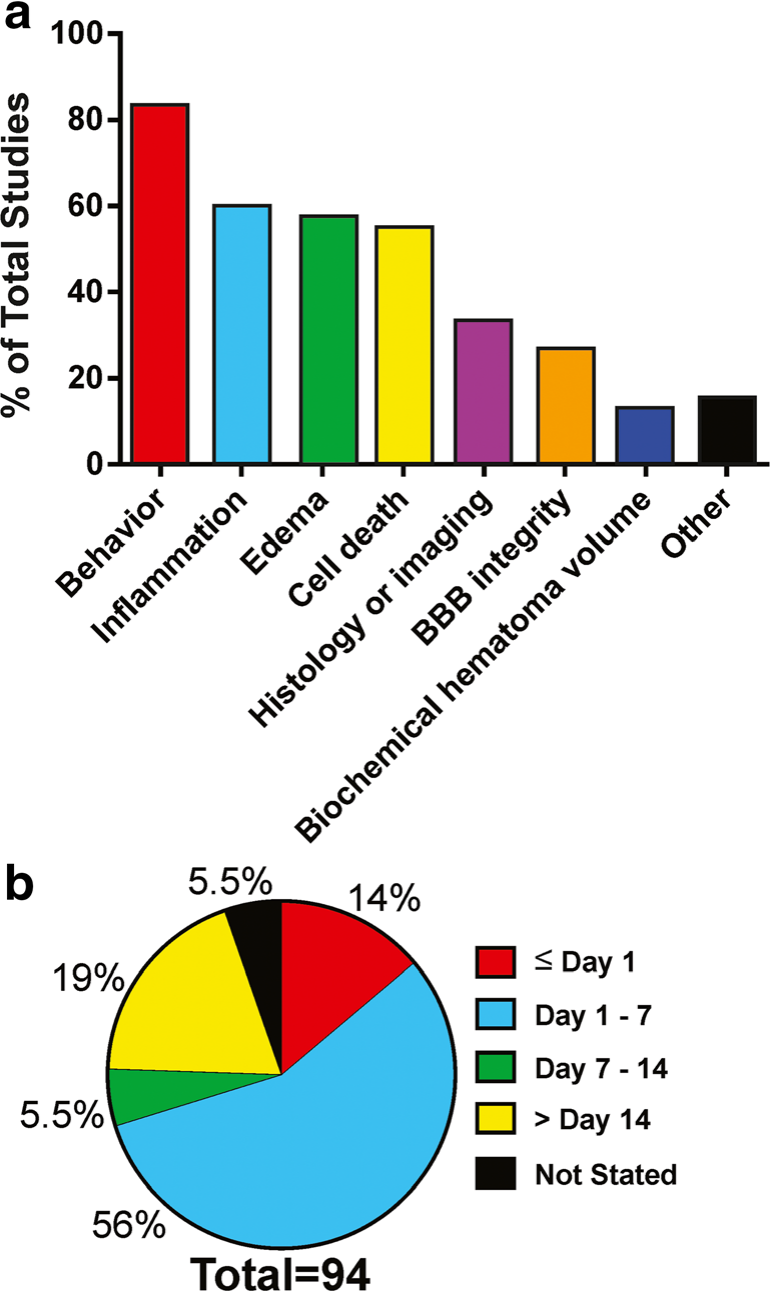
Analysis of tissue endpoints used in ICH neuroprotection research. **a** Categorization of endpoints into classes. **b** Latest timing of injury volume assessment

For an overview of tissue endpoints in preclinical ICH literature from 2015 to 2019, see Fig. 4a. Overall, we found that most studies used endpoints related to the reduction of inflammation (~ 60%), edema (~ 58%), and cell death (~ 55%). Fewer studies assessed blood brain barrier (BBB) disruption or hematoma volume.

#### Injury Volume Estimation and Latest Timing of Assessment

Ninety-four studies (~ 33% of 281 studies) performed histological or imaging assessment of injury volume. To look at the timing of the injury volume assessment, we collected the latest assessment time in each study. We categorized the studies into those where the assessment was on or before day 1 post-ICH, between 1- and 7-day post-ICH, between 7- and 14-day post-ICH, and studies which assessed injury volume later than 14-day post-ICH. Of the 94 studies that performed injury volume assessment, 89 reported when the assessment occurred. The results of our analysis are in Fig. 4b. We found that 14% of studies assessed injury volume on or before day 1 post-ICH. A majority of studies (56%) performed injury volume assessments between days 1 and 7 post-ICH. About 19% of studies that assessed lesion size did so at a long-term survival time (> 2-week survival). Thus, only ~ 6% of all studies assessed long-term injury volume.

### Behavioral Tasks and Timing of Assessment

**Fig. 5:**
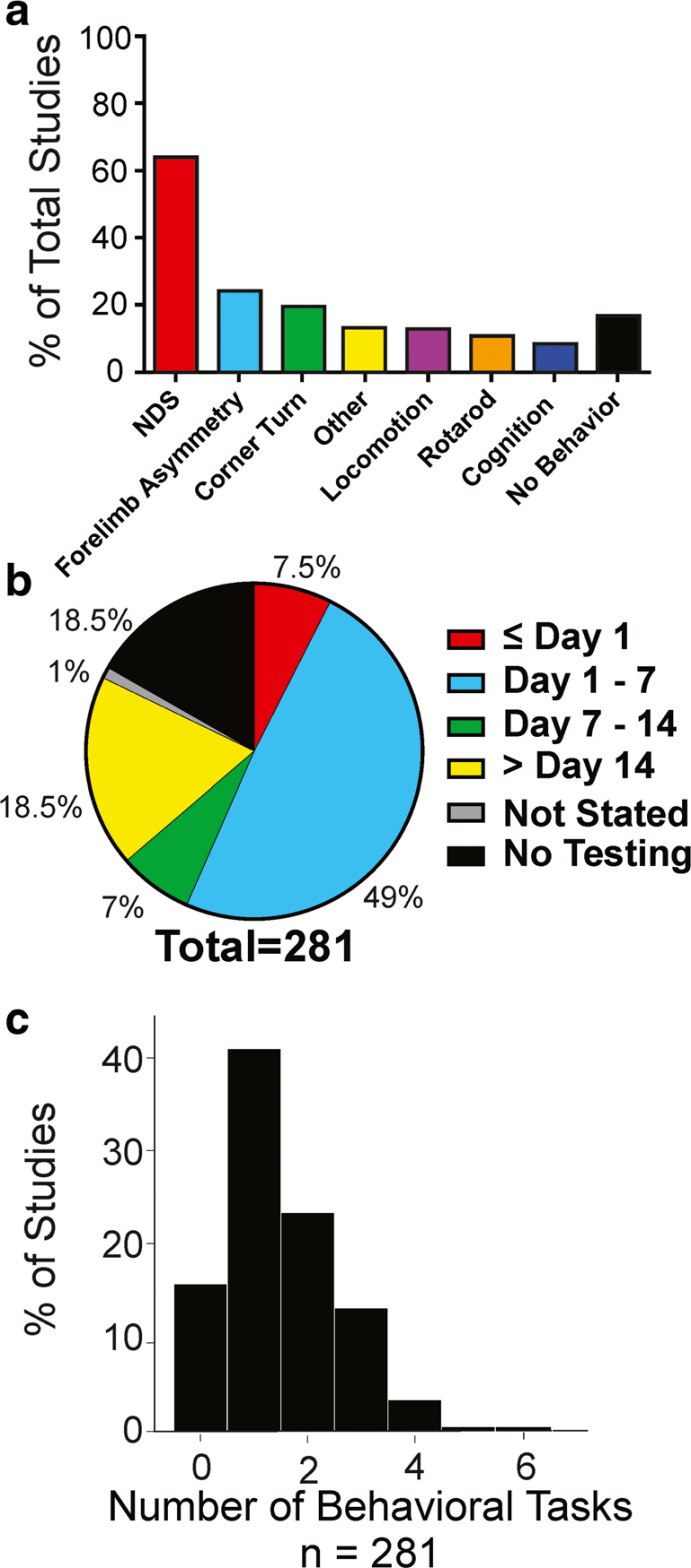
Assessment of behavioral endpoints used in ICH neuroprotection research. **a** Proportion of behavioral endpoint use. Neurological deficit scales are common endpoints in ICH neuroprotection research. **b** Latest time of behavioral testing for all studies. A majority of studies conduct behavioral testing on or before day 7 post-ICH. **c** Number of behavioral tasks used per study. About 50% of current ICH neuroprotection research make use of 2 or more behavioral tests per study

Similar to our analysis of tissue endpoints, we looked at the prevalence of various behavioral tasks used in the assessment of post-ICH functional outcomes and the timing of these assessments. Overall, 47 studies did not perform behavioral assessment, and 234 studies used 1 or more behavioral tests. We found heavy reliance on a variety of neurological deficit scales (NDS) to gauge behavioral outcomes following ICH (Fig. 5a**)**. The next most common assessments included forelimb use asymmetry (i.e., cylinder or forelimb placing tasks), followed by corner turn assessments. The “other” category included assessments of somatosensation, proprioception, and additional uncommon assessment strategies.

#### Timing of Latest Behavioral Assessment

Of the 234 studies which reported using behavioral assessments, 231 clearly reported the timing of behavioral assessment. Again, we collected data with respect to timing of latest behavioral assessment. The results are in Fig. 5b. Of the studies that conducted behavior, we found that most studies (77.5% of 231) conduct assessments on or before day 7 post-ICH and 22% of studies conduct long-term (> day 14) behavioral assessments. We also found that about 50% of published ICH neuroprotection studies make use of 2 or more behavioral tests per study (range = 1–6; Fig. 5c). Out of all studies, 18.5% conducted long-term behavioral testing.

### Largest Reported Group Size

**Fig. 6:**
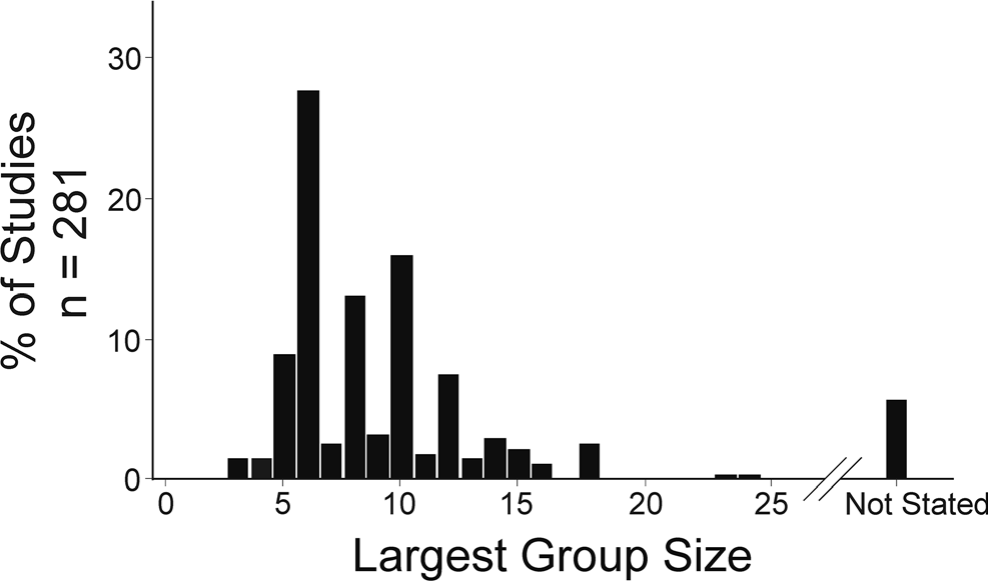
Analysis of largest reported group sizes in experimental ICH research (bin width = 1). Most studies use 6 animals per group as their largest group sizes

Because few studies (~ 12.5%) reported using a priori group size calculations to select group sizes, we assessed group sizes used in published research. By looking at the largest group sizes in each study, we found a skewed distribution, ranging from 3 to 24 animals per group (mean = 8.6, SD = 3.5; median = 8, IQR = 6–10). A histogram of the largest reported group sizes can be found in Fig. 6.

We were also interested in whether group sizes used in a study were increased when a priori sample size calculations were performed. We found that studies performing a priori sample size calculations had larger group sizes (~ 2 more animals per group) when compared with studies that did not perform these calculations (*p* = 0.0092).

#### Publication Venue Characteristics

**Fig. 7:**
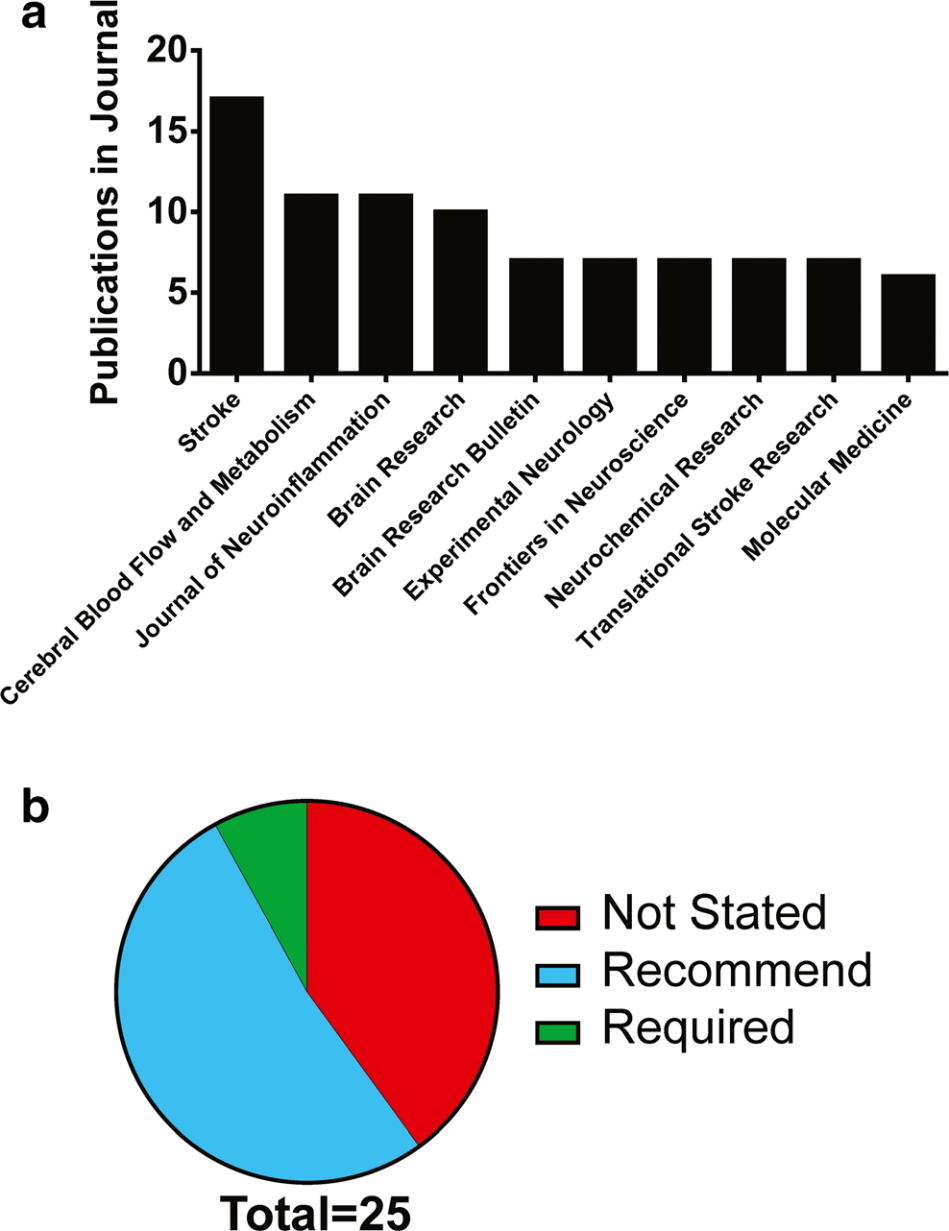
Analysis of most common publication venues for preclinical ICH neuroprotection research. **a** The top 10 most popular journals from 2015 to 2019 and the number of ICH neuroprotection papers published in each. **b** Analysis of the top 25 journals that publish ICH neuroprotection research and whether experimental design and reporting in accordance with published guidelines was mandatory, recommended, or not mentioned

We found that the most popular journals used for publishing ICH neuroprotection research were *Stroke*, the *Journal of Cerebral Blood Flow and Metabolism*, and the *Journal of Neuroinflammation*, respectively (Fig. 7a). Publications in the most common 3 journals accounted for 13% of the total publications analyzed. Publications in the top 25 most popular journals accounted for about 50% of all publications analyzed. We analyzed the top 25 most popular journals for statements related to mandatory reporting in accordance with experimental design and reporting guidelines and found that 2 journals required mandatory publication of experimental design characteristics according to published guidelines in the form of a checklist as supplemental material (Fig. 7b). Fifty-two percent of journals recommended reporting in accordance with published guidelines, and 40% of journals did not mention reporting or designing experiments in accordance with published guidelines.

## Discussion and Additional Considerations

Developed with the “3 R’s” of animal ethics in mind, the ARRIVE guidelines aimed to improve the reporting standards for animal experiments [28, 32]. Recently, ethicists have proposed a “fourth R”: reproducibility [7, 33, 34]. The authors of ARRIVE sought to reduce the costs and consequences of incomplete study reporting [28]. Similarly, in stroke neuroprotection, several guidelines intend to enhance reproducibility and reduce bias [9–11]. Here, we used those guidelines as a framework for analyzing ICH neuroprotection studies. Generally, reporting simple experimental design features is commonplace, but imperfect. Published guidelines can improve research quality, especially when adopted and enforced by editors, journals, and funders [28, 35, 36]. Altogether, guidelines can benefit authors (by making a clear process), publishers (by creating higher quality publications), and readers (by maximizing information available) [28, 37].

In 2012 we investigated experimental quality in preclinical ICH research [22]. There have been some improvements since then, but many aspects are similar. Key changes from that study include a large increase in use of chloral hydrate, greater use of mice, and a greater prevalence of cell death and inflammation measurements. Interestingly, our previous study was published closely after the publication of the ARRIVE guidelines, so some improvements were expected, though more work must be done. Finally, authors in subarachnoid hemorrhage noted transient improvements in experimental reporting following the publication of the ARRIVE guidelines, suggesting that system-wide, collaborative changes must be implemented to reach and sustain a standard of experimental design reporting [38].

To briefly summarize our findings, almost all studies use young, healthy, male rodents. Authors frequently provide weight information to refer to the developmental status of their animals and infrequently provide age information; both are important [28]. Indeed, age may not be accurately gleaned from weight (e.g., due to species and strain differences, husbandry conditions, if food restriction is used) [39–41]. Few studies use animals with advanced age or comorbidities. Studies considering these conditions are critical to understanding treatment efficacy, as most ICH patients are not young and healthy. Indeed, many have shown decreased treatment efficacy with stronger adherence to translational design [15]. Echoing this, a recent meta-analysis of preclinical ischemia studies could not find evidence for therapeutic efficacy across many treatments in studies using animals with comorbidities [42]. Lastly, many clinical studies do not find sex differences in the incidence of ICH [3, 43]. Despite this fact, almost all ICH research uses male animals. Moreover, sex differences in post-ICH outcomes are understudied, despite the expectation that they should be considered [44–46]. Previous research has shown that sex hormones (e.g., estradiol) affect post-ICH injury trajectories [47, 48]. Unfortunately, we found that very few studies use female rats. Future preclinical ICH studies should consider biological sex, and calls are being made to investigate these issues [14, 44–46]. Although animal models of ICH appear relatively homogenous, our findings may underestimate the heterogeneity of translational design since no single group would assess every endpoint and parameter in the short and long term [49].

Investigators must consider the use of anesthetics and analgesics, as they can alter post-ICH injury trajectories [50, 51]. Indeed, the most common 3 anesthetics identified here have significant concerns. For example, pentobarbital, chloral hydrate, and isoflurane affect thermoregulation, and some can decrease body temperature for hours despite providing external heating [51–53]. Although the relationship between brain damage and temperature is better known in other brain injuries, anesthetic-driven hypothermia should be avoided in ICH [51]. Additionally, general anesthetics depress respiration. Finally, general anesthesia can reduce blood pressure which may modify post-ICH outcomes, particularly in the collagenase model where bleeding occurs over hours [26, 54, 55]. Little is known about the impact of analgesics on post-ICH outcomes. For example, one study showed that buprenorphine decreased hematoma volume while increasing peri-hematoma cell death in the collagenase model [50]. Buprenorphine also affects inflammation and suppresses respiration at high doses [56]. Interestingly, chloral hydrate is the most commonly used anesthetic (Fig. 2b), but its analgesic properties are inadequate, and the agent is toxic [57–60]. These issues are relevant, especially for multiple surgical preparations and long-term survivals. We recently demonstrated that ICH can be induced in awake freely behaving animals using collagenase; this method may avoid anesthetic-related confounds [51]. Altogether, analgesics and general anesthetics may alter many physiological processes, leading to experimental confounds if improperly considered.

Group size reporting is ubiquitous in preclinical ICH studies, but the method of determining group sizes is rarely mentioned. Studies using a priori sample size calculations generally had larger group sizes than those that did not. Notably, we considered only the *largest* group sizes used in a study, and often, group sizes for other endpoints were smaller. Thus, future reports should detail the group size calculation for all endpoints [10, 11, 28]. Group sizes are a critical issue, and they present researchers with an ethical dilemma because researchers are obligated to use the fewest animals possible [32]. Conversely, researchers must ensure that they have sufficient statistical power. Indeed, conclusions may be impossible to draw from an underpowered study [10, 61, 62]. A priori sample size calculations may solve this dilemma, as they represent the fewest animals necessary to detect an expected effect.

Most ICH studies use neurological deficit scoring, forelimb use asymmetry tasks, or the corner turn test, which are simple and quick to administer. Although deficit scales appear to mimic clinical assessments, these tasks are often not sensitive to chronic impairments [4, 63]. These scales also involve subjective assessments, and thus, blinded assessments are critical. However, these tests are reasonable to use as a gross assessment strategy and possibly in tandem with tasks that are sensitive to chronic behavioral deficits. Indeed, we and others have found that a battery of tests discriminates ICH injury better than any single test and thus researchers should use multiple tests and assessment times longer than 2 weeks whenever possible [4, 10, 63, 64].

Our analysis of brain tissue endpoints revealed that most assess inflammation, edema, or cell death. Inflammation was frequently measured with immunohistology or western blotting. Edema was mostly assessed with wet-dry measurements. Cell death was mostly measured using TUNEL or Fluoro-Jade counts. Generally, tissue endpoints in preclinical research are difficult to translate to clinic, and thus calls have been made to investigate biomarkers that predict neuroprotective efficacy [4]. While preclinical research is advantageous because mechanisms can be evaluated, they must still be established as biomarkers (i.e., shown on a translatable scale, alongside neurological improvements), as described by the STAIR/RIGOR guidelines [4, 9, 14].

We began this study with the premise that long-term histological or imaging assessments of injury may be easily translatable endpoints in preclinical research. This is because neuroimaging is the gold standard for assessment and diagnosis of ICH and these data is easily available [65]. Additionally, many studies have determined the impact of injury volume and location on death and disability in animals and humans [63, 66–69]. We also reasoned that short-term injury assessments produce an incomplete picture, as injury occurs for weeks [26, 27, 70]. Moreover, mass effect complicates short-term injury assessments, which can result in biased measurements if tissue displacement is not considered. Moreover, cell death assessments may not be feasible in a long-term study and are influenced by the region of interest. Conversely, long-term injury volume assessments capture the end-product of injury and repair processes that result in an easily defined cavity without mass effect and are feasible owing to low mortality rates in preclinical models [71, 72]. Although other tissue endpoints are informative, we propose (in line with published guidelines) that long-term preclinical injury volume measurements are translationally valuable as they are established biomarkers [10]. However, we acknowledge that depending on the preclinical model used, there may be key differences in cerebral anatomy that must be considered (e.g., lissencephaly).

Lastly, to better understand the context of current research, we analyzed the most popular publishing venues and whether they require that manuscripts comply with experimental design and reporting guidelines. Few journals require mandatory compliance with published guidelines. About half of the most popular journals suggest reporting in accordance with ARRIVE or similar guidelines, but do not mandate it. Finally, 40% of the most popular journals in ICH do not recommend or require alignment with published guidelines. These facts are concerning given calls for transparent, translationally rigorous preclinical research [5, 9, 11, 28, 49].

### Can We ARRIVE at a Standard for Experimental Design and Reporting by Putting our HEADS Together?

In 2018, the Hemorrhagic Stroke Academia Industry (HEADS) Roundtable published a report addressing current practices, priorities, and limitations of translational ICH research [73]. Several of our recommendations overlap with those, which are presented in Table 1. For example, the HEADS report stressed that advanced age and comorbidities must be addressed, speculating that most preclinical ICH research is performed in young, healthy animals, which our results confirm. Studies that consider advanced age and hypertension are important as they are established risk factors for ICH incidence and recurrence in patients [2, 74].

**Table 1:** Overlapping recommendations for contemporary preclinical ICH research and adherence to recommendations

Recommendation	% Adherence
Neuroprotective efficacy based on improvement on established biomarkers (e.g., behavior and histology) over extended survival times. Improvements on other endpoints should not be accepted as evidence of neuroprotection without proof of their translational relevance	Behavior: 83.3% Histology: 33.5% Behavior and histology: 29.9%
Use of a realistic treatment delay (e.g., > 3 h)	23.1%
Use of a priori sample size calculations	12.5%
Use of multiple models (multiple ICH models and/or multiple species)	6.4%
Use of aged animals	3.2%
Use of male and female animals	3.2%
Use of animals with comorbid conditions	2.5%
Efficacy shown across a variety of injury sizes	0%

Readers are also referred to the recent HEADS publication [73]

Similarly, HEADS recommended finding biomarkers to guide neuroprotective interventions. Our results suggest that most authors consider inflammation, cell death, and edema to be important ICH biomarkers. We found a heavy focus on preventing these injury processes. However, many post-stroke processes are multiphasic [73, 75]. Thus, timing of treatment onset should be considered, but few investigated multiple treatment delays, and most administered treatments before or immediately following ICH. Relying upon hyperacute treatments means that most ICH neuroprotection studies do not consider realistic therapeutic windows, as patient studies often show treatment delays of several hours [76–78]. Also, in the collagenase model, treatments administered in the first few hours must be considered as occurring during hematoma formation and are therefore more relevant to a smaller portion of re-bleeding patients that receive very early treatment. In sum, more work must be done to evaluate parameters under which neuroprotectants are effective (e.g., efficacy following a clinically relevant treatment delay).

To best address the issues raised here and in the HEADS report, a common recommendation is to systematically form networks that engage in high-quality ICH research. Indeed, Table 1 suggests that at the individual level, many of the HEADS recommendations are not being met. Although one group probably cannot assess every translational issue, several laboratories may [4, 73]. The HEADS report recognizes that ARRIVE and STAIR guidelines must be followed and recognize the value of replication studies. Authors may resist running replication studies, with concerns that the study may not replicate. These concerns must subside, as scientific inquiry must be supported by rigor and replication. Moreover, it is unethical to leave “unsuccessful” replication studies unpublished [9, 14, 73]. Several journals recognize the value of “negative” studies, and avenues exist to publish studies before they are conducted (i.e., registered studies). We and many others maintain that both “positive” and “negative” studies are essential to translational success, as they contribute to the evidence-base of a therapy [7, 42, 79, 80].

### Conclusion and Summary

We conducted this study to assess research design and reporting in current preclinical ICH research, recognizing that inappropriate or inadequate research design can produce biased research findings and hinder translational success [9, 10, 15, 28]. We found that reporting of basic design elements has become commonplace, but there is significant room for improvement in terms of both experimental reporting and design. We note that published guidelines exist to aid investigators in research development and dissemination [9, 10, 28, 73]. Finally, publishers infrequently require mandatory reporting of critical experimental characteristics. In order to see improvements, publishers must require reporting in accordance with published guidelines, as the mere publication of guidelines without their enforcement may not improve reporting [38]. A collaborative approach towards improved experimental design and reporting is suggested at all levels of the publication process.
